# Using iNaturalist to monitor adherence to best practices in bat handling

**DOI:** 10.3897/BDJ.9.e68052

**Published:** 2021-09-23

**Authors:** Laura Van der Jeucht, Quentin Groom, Donat Agosti, Kendra Phelps, DeeAnn Marie Reeder, Nancy B. Simmons

**Affiliations:** 1 Free University of Brussels (VUB), Brussels, Belgium Free University of Brussels (VUB) Brussels Belgium; 2 Meise Botanic Garden, Meise, Belgium Meise Botanic Garden Meise Belgium; 3 Plazi, Bern, Switzerland Plazi Bern Switzerland; 4 EcoHealth Alliance, New York, United States of America EcoHealth Alliance New York United States of America; 5 Bucknell University, Lewisburg, United States of America Bucknell University Lewisburg United States of America; 6 American Museum of Natural History, New York, United States of America American Museum of Natural History New York United States of America

**Keywords:** anthropozoonosis, personal protective equipment, handling animals, safety

## Introduction

The general guidance is, and has always been, that handling bats should be avoided, particularly by the general public, but capturing and handling bats is often unavoidable for bat researchers. While bat researchers are aware of the potential for zoonotic pathogen transmission to occur when handling bats, most notably Rabies virus, some do not wear any (or insufficient) personal protective equipment (PPE) to reduce risks of exposure. This lack of adherence to even minimal biosafety practices may jeopardize both the safety of the bat and the handler. Such concerns became more pressing with the COVID-19 pandemic, but also had been raised as a result of previous outbreaks of human infections linked to contact with animals. The largely unknown potential for handled bats to become infected by a bat researcher, something not previously considered by most field workers, is now widely known in the research community due to the efforts of the IUCN SSC Bat Specialist Group and the Global Union of Bat Diversity Networks (GBatNet).

It is also worth noting that the negative framing of bats during the pandemic may have serious consequences for bat conservation (López-Baucells et al. 2017) and a refocusing of the conversation on the deficiencies of human interactions with bats would be a preferable direction. After all, bats are an important component of global ecosystems and provide many ecosystem services to humans (Kunz et al. 2011).

Several bat-specific conservation groups have developed guidelines on how to safely handle bats to prevent anthropozoonotic transmission, including the IUCN SSC Bat Specialist Group (Nuñez et al. 2020), the Bat Conservation Trust (Anonymous 2020), the Latin American and Caribbean Network for the Conservation of Bats (Suárez-Alvarez and López-Berrizbeitia 2020) and Worksafe Queensland (Paterson 2016). These guidelines largely focus on the increased use of PPE, such as leather or nitrile/latex gloves, respirator masks, long sleeves and long trousers and protective eyewear or face shield, depending on the situation. These build on earlier PPE protocols deployed by some research groups to mitigate the risk of exposure to potential bat pathogens.

Given the recent developments, we were interested to know if the use of PPE, in particular wearing gloves, has improved and whether we could detect a change by screening pictures of bats uploaded to iNaturalist. Clearly, it is not possible to evaluate the full adherance to bat handling guidlines, but we believe that use of gloves is a good indicator that best practices are being followed.

iNaturalist is a mobile phone app for recording occurrences of wildlife and a social network for amateur and professional naturalists alike. It has about 100,000 to 300,000 active users and tens of thousands of observations of wildlife are recorded each day. On iNaturalist, most observations are evidenced by a picture of the organism. This allows the species to be identified, if possible, or verified by users who have the expertise, but also provides the context of the photograph as well. That context can show what the organism is doing, what developmental stage it is, what the habitat is or how it was photographed, such as whether a person is holding the organism. In our case, we wanted to verify whether people who were handling bats were wearing gloves and to determine whether there has recently been a change in behaviour (i.e. frequency of bat handlers wearing gloves).

## Methods

We evaluated photographs of bats submitted between 2008 to 2021 out of a total of 43,561 Chiroptera observations available on iNaturalist as of 1 February 2021. From these, we screened photographs until we had found 1000 photographs in which a person was clearly depicted handling a bat, whether dead of alive. About 15% of all observations fitted these criteria. Each observation was scored on whether or not the handler was wearing gloves. Cases, in which only one hand was gloved or a glove was ripped, were treated as if the handler was not using gloves. If the person was holding the bat with a holding bag or some other fabric separating the bat from the skin of the handler, we treated this as equivalent to gloved handling as long as we could not see the other hand in the photograph. The licence for each observation was checked and only openly-licensed data were analysed. This removed 239 observations. Seven hundred and fifty nine of the observations had open coordinates for the observation and these are mapped. It is noted that iNaturalist began in 2008, but contains earlier observations because pre-existing photographs are uploaded. Therefore, we were able to include observations of bats dating back to the 1980s.

Data on individual observations were extracted from iNaturalist using the 'rinat' package (Barve et al. 2021). The results were fitted using logistic regression with date as the independent variable in R version 3.6.1 (R Core Team 2014). All data and code to generate the statistics and figures are available on Zenodo (Groom 2021).

## Results

**Figure 1.**
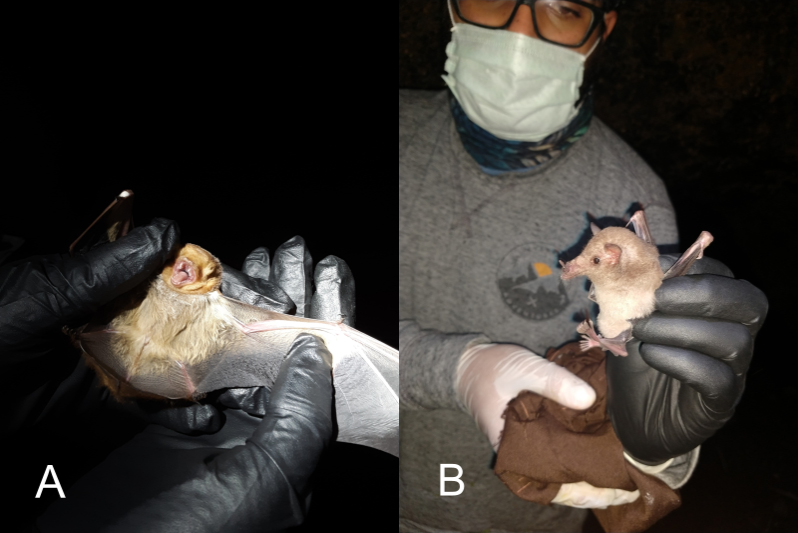
Examples of appropriate use of personal protective equipment when handling bats. A. An eastern red bat (*Lasiurusborealis*) handled with gloves (July 2020; https://www.inaturalist.org/observations/62466174), ©Brayden Paulk (CC-BY-NC). B. An example of bat researcher holding a long-tongued bat (*Choeronycterismexicana*) with gloves, eye protection and a face mask (September 2020; https://www.inaturalist.org/observations/60339444), © Axl Hernández (CC-BY-NC).

**Figure 2.**
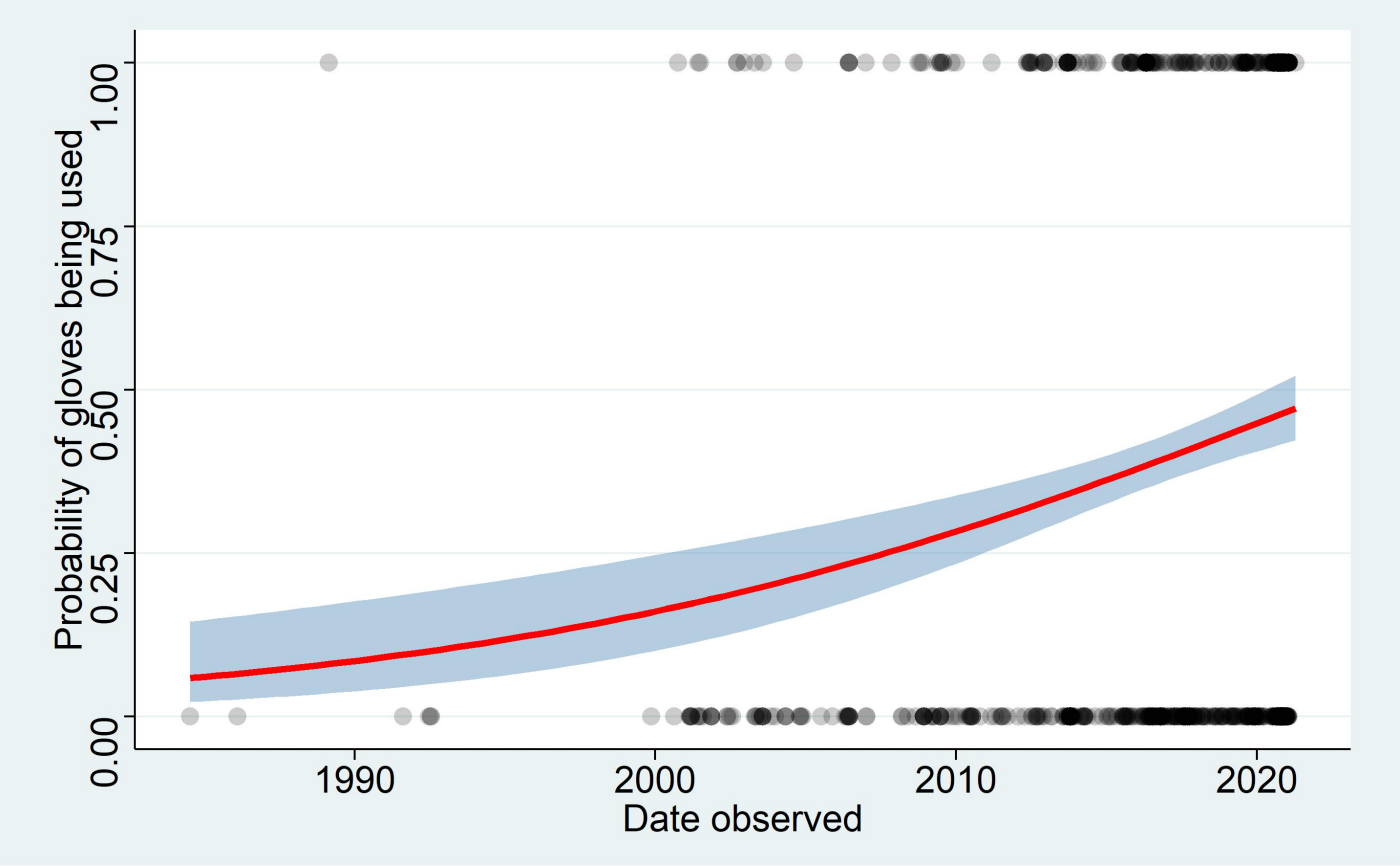
The probability of glove usage when handling bats as inferred from 761 photographs uploaded to iNaturalist between 2008 and 2021 that included a clear depiction of the observer handling a bat and were openly licensed. The shaded area is the 95% confidence interval.

**Figure 3.**
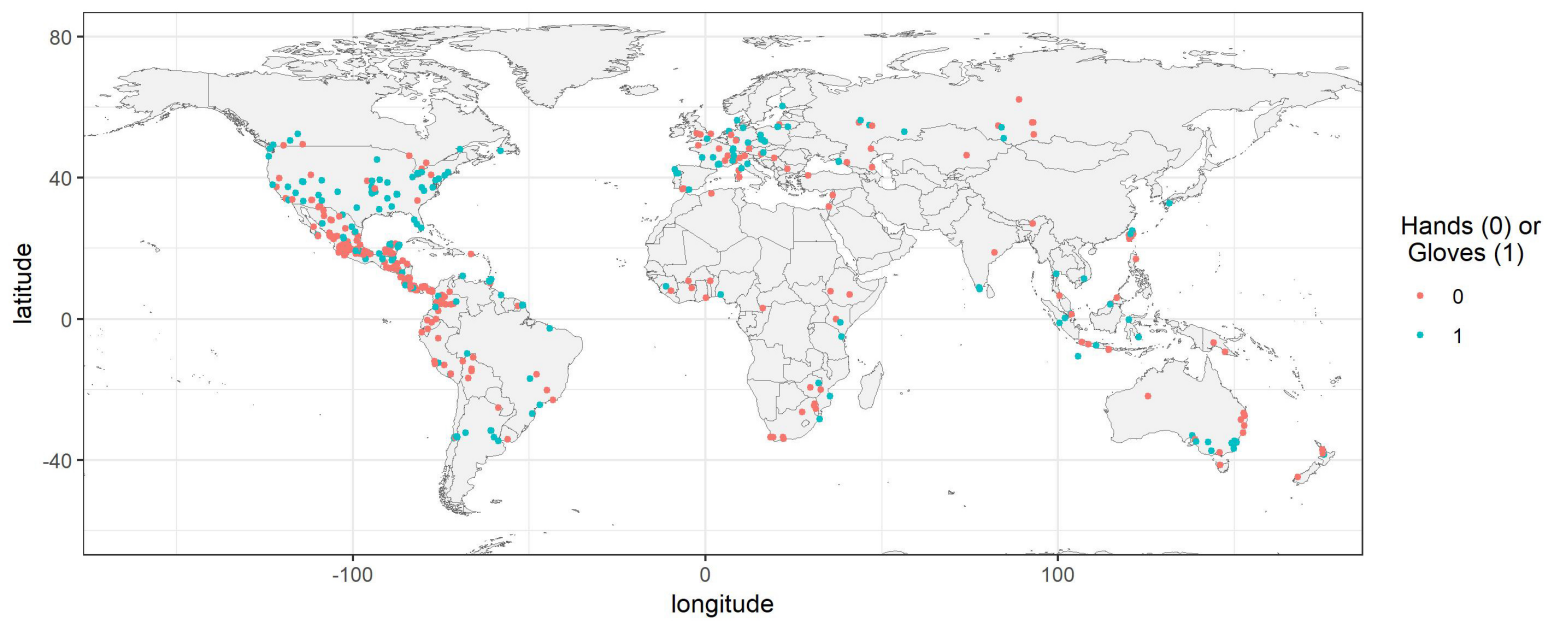
Distribution of iNaturalist observations where gloves were or were not used while handling bats.

There are many examples of appropriate use of PPE in photographs showing bats on iNaturalist (Fig. 1). However, glove wearing has increased over the past two decades (Fig. 2). The observations are concentrated in Europe, North America and South America, as are most iNaturalist records (Fig. 3). However, there is no obvious spatial trend where gloves are being used. The results of the logistic regression are shown in Table 1. The number of iNaturalist observations has been increasing over the period of the study, so the confidence interval has improved with time. Amongst the users who submitted these observations, 165 only ever used gloves, 214 only ever used bare-hands and only 38 had observations where sometimes they used gloves.

## Discussion

Bats are implicated as reservoir hosts for a wide variety of potentially zoonotic viruses, including filoviruses, henipaviruses, lyssaviruses and coronaviruses (Anderson et al. 2019, Drexler et al. 2014, Olival and Hayman 2014). Yet, it is rare that contact with bats is fatal to humans; it is probably more often fatal to the bat due to injury or stress from being handled. Indeed, white-nose disease pathogen (*Pseudogymnoascusdestructans*) has killed many millions of bats in North America and cave visitors are possibly unwitting vectors (Zhelyazkova et al. 2020). Now researchers are worried that humans may also potentially pass their own pathogens to bats. SARS-CoV-2 has a wide host range (Hossain et al. 2021, Schlottau et al. 2020) and could potentially be transmitted from humans to bats (Gryseels et al. 2020, Schlottau et al. 2020, Cook et al. 2021). This could not only endanger the health of the bat, but could also lead to transmission amongst bats and establishment of SARS-CoV-2 in bat populations, possibly allowing the virus to mutate and eventually potentially be transmitted back to humans as a new variant. More research is needed on the taxonomy, evolution and ecology of bats, and handling bats is unavoidable for many researchers. In our sample from iNaturalist, not all handlers may have been trained bat researchers and many may not have been aware of the risks associated with unprotected contact with wild animals. It, therefore, appears that more needs to be done to educate people before use of PPE becomes universal. There does not seem to be a geographic trend to the incidence of people wearing gloves while handling bats, which suggests that the need for training and awareness raising is global.

On the 11 March 2020, the World Health Organisation announced that COVID-19 could be considered a pandemic. However, there is little evidence in the data that there has been a major change in the probability of people using gloves when handling bats; rather, there has been a gradual increase in use of gloves while handling bats over the course of the last 20 years, presumably as awareness of zoonosis has increased. Clearly, it takes time for changes in practice to filter though a research community. Many bat researchers have not been able to conduct fieldwork since the beginning of the pandemic, but when they do go back in the field, many more are likely to be wearing gloves, especially after the IUCN Bat Specialist Group issued guidelines in 2020 stressing the importance of this practice. The data and code to recreate and add to this analysis have all been made publicly available (Groom 2021) and it would be interesting to add to the current analysis in a year or two to see if there is significant change in bat handling practices post-pandemic.

In addition to demonstrating changes to bat handling techniques, this paper demonstrates yet another use for the images shared on platforms, such as iNaturalist for research related to biodiversity. These photographs are important evidence for validating observations, but also contain a large amount of contextual information about the organism and its surroundings. For example, photographs, such as those on iNaturalist, have been used to study the phenology of plants (Barve et al. 2020) and emergence of insects (Jain and Tea 2020). Data on biotic interactions can be extracted from photos (Seltmann et al. 2020, Gazdic and Groom 2019, Naude et al. 2019) and they can also be used to determine physical traits of organisms (Drury et al. 2019). Indeed, it is said that "*A picture is worth a thousand words*" and in truth, the photographs on iNaturalist encapsulate much more information than is textualised in the associated metadata. Furthermore, although we extracted information from iNaturalist photographs manually, there is clearly scope to use machine learning to extract data in much larger volumes in future projects.

## Figures and Tables

**Table 1.** Results of the logistic regression of the use of gloves when handling bats (i.e. gloves or bare-hands) in the last two decades.

	**Logistic regression coefficient**	**Standard Error**
(Intercept)	-3.818	0.73***
Observation date	1.98e-04	4.26e-05***
